# MetaNetX.org: a website and repository for accessing, analysing and manipulating metabolic networks

**DOI:** 10.1093/bioinformatics/btt036

**Published:** 2013-01-28

**Authors:** Mathias Ganter, Thomas Bernard, Sébastien Moretti, Joerg Stelling, Marco Pagni

**Affiliations:** ^1^Department of Biosystems Science and Engineering, ^2^SIB Swiss Institute of Bioinformatics, ETH Zurich, 4058 Basel, Switzerland, ^3^Vital-IT, SIB Swiss Institute of Bioinformatics, 1015 Lausanne, Switzerland and ^4^Department of Ecology and Evolution, University of Lausanne, 1015 Lausanne, Switzerland

## Abstract

**Summary:** MetaNetX.org is a website for accessing, analysing and manipulating genome-scale metabolic networks (GSMs) as well as biochemical pathways. It consistently integrates data from various public resources and makes the data accessible in a standardized format using a common namespace. Currently, it provides access to hundreds of GSMs and pathways that can be interactively compared (two or more), analysed (e.g. detection of dead-end metabolites and reactions, flux balance analysis or simulation of reaction and gene knockouts), manipulated and exported. Users can also upload their own metabolic models, choose to automatically map them into the common namespace and subsequently make use of the website’s functionality**.**

**Availability and implementation:** MetaNetX.org is available at http://metanetx.org.

**Contact:** help@metanetx.org

## 1 INTRODUCTION

Genome-scale metabolic networks (GSMs) consist of compartmentalized reactions that consistently combine biochemical, genetic and genomic information. When also considering a biomass reaction and both uptake and secretion reactions, GSMs are often used to study genotype–phenotype relationships, to direct new discoveries and to identify targets in metabolic engineering (Karr *et al.*, 2012). However, a major difficulty in GSM comparisons and reconstructions is to integrate data from different resources with different nomenclatures and conventions for both metabolites and reactions. Hence, GSM consolidation and comparison may be impossible without detailed biological knowledge and programming skills. Therefore, community approaches in form of jamboree meetings were introduced to collect and integrate data to generate consensus reconstructions (Herrgaard *et al.*, 2008). Furthermore model repositories, such as BiGG (Schellenberger *et al.*, 2010), MetRxn (Kumar *et al.*, 2012) or the Model SEED (Henry *et al.*, 2010) were developed to integrate models and to allow comparative analyses. In addition, tools like the COBRA Toolbox (Becker *et al.*, 2007), CytoSEED (DeJongh *et al.*, 2012), FAME (Boele *et al.*, 2012) or OptFlux (Rocha *et al.*, 2010) assist in the analysis and modelling tasks. However, a tight integration of models and software is currently only provided by the Model SEED, and most of the advanced tasks, like model manipulations (reaction direction assignment, adding or removing candidate reactions, modifying the objective function), are limited to experienced users with programming skills.

## 2 OVERVIEW

**Fig. 1.**
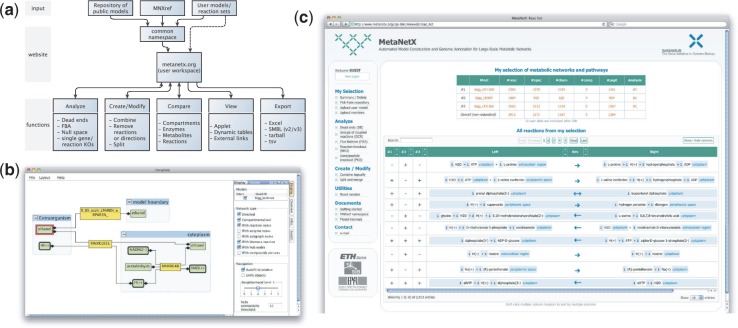
Main features of MetaNetX.org. (**a**) Flowchart illustrating the structure of MetaNetX.org: the website with its common namespace and user workspace connects the repository of publicly available GSMs or user-defined GSMs/reaction sets with the tools section, as well as the view and export functions that enable the users to interactively analyse their results inside or outside of MetaNetX.org. (**b**) Interactive applet viewer showing the network neighbourhood of ethanol across several compartments in a selected *Escherichia coli* model. (**c**) Comparison of three selected *E. coli* models (top) using the MetaNetX.org workspace: present (+) or absent (−) reactions (bottom)

MetaNetX.org is implemented as a user-friendly and self-explanatory website that handles all user requests dynamically (Fig. 1a). It allows a user to access a collection of hundreds of published models, browse and select subsets for comparison and analysis, upload or modify new models and export models in conjunction with their results. Its functionality is based on a common namespace defined by MNXref (Bernard *et al.*, 2012). In particular, all repository or user uploaded models are automatically translated with or without compartments into the common namespace; small deviations from the original model are possible due to the automatic reconciliation steps implemented by Bernard *et al.* (2012). However, a user can choose not to translate his model but still make use of the website’s functionalities. Furthermore, it is possible to augment the given reaction set by user-defined reactions, for example, for model augmentation.

Any available network or pathway can be examined at metabolite, reaction, enzyme, pathway or compartment levels using, for example, an interactive graphical user interface [in contrast to static KEGG maps (Kanehisa *et al.*, 2012); Fig. 1b] or information provided at UniProt/SwissProt. In addition, two or more GSMs or pathways (even from different resources like BiGG, MetRxn or UniPathway) can be compared to determine (un)common parts (Fig. 1c).

MetaNetX.org also offers an extensive tools section for analyses based on the network structure (stoichiometric matrix) or on flux balance analysis (Gianchandani *et al.*, 2010). Specifically, it offers services to identify structural inconsistencies such as dead-end metabolites and their affected (downstream) reactions and metabolites as well as zero-flux reactions and inconsistent correlation groups (Terzer *et al.*, 2009). For simulations, MetaNetX.org provides a tool set to study reaction fluxes, in particular with respect to biomass production and biomass production after performing single reaction or single gene knockouts, which are commonly used for model validation.

In the context of model development, a dedicated section of MetaNetX.org allows one to combine GSMs with respect to their reaction or protein sets or with respect to the results of previously performed analyses. For example, it is possible to create a minimal functional model where every reaction is able to carry a flux at steady state, i.e. a model without zero-flux reactions.

All available and newly generated networks as well as the results of their analyses and predictions can be exported as SBML- or flat-files for documentation and further analyses/modifications in external tools such as the COBRA toolbox (Becker *et al.*, 2007).

We believe that MetaNetX.org could become a valuable resource for accessing, analysing and manipulating GSMs, especially for users with limited programming skills, or as a resource for independent validation and testing. We expect that the rigorous format requirements enable a standardized way to define and exchange models and that they allow for an effective and efficient benchmark process for future method development projects.

## References

[btt036-B1] Becker SA (2007). Quantitative prediction of cellular metabolism with constraint-based
models: the COBRA Toolbox. Nat. Protoc..

[btt036-B2] Bernard T (2012). Reconciliation of metabolites and biochemical reactions for metabolic
networks. Brief. Bioinform.

[btt036-B3] Boele J (2012). FAME, the flux analysis and modeling environment. BMC Syst. Biol..

[btt036-B4] DeJongh M (2012). CytoSEED: a Cytoscape plugin for viewing, manipulating and analyzing
metabolic models created by the model SEED. Bioinformatics.

[btt036-B5] Gianchandani EP (2010). The application of flux balance analysis in systems biology. Wiley Interdiscip. Rev. Syst. Biol. Med..

[btt036-B6] Henry CS (2010). High-throughput generation, optimization and analysis of genome-scale
metabolic models. Nat. Biotechnol..

[btt036-B7] Herrgaard MJ (2008). A consensus yeast metabolic network reconstruction obtained from a
community approach to systems biology. Nat. Biotechnol..

[btt036-B8] Kanehisa M (2012). KEGG for integration and interpretation of large-scale molecular data
sets. Nucleic Acids Res..

[btt036-B9] Karr JR (2012). A whole-cell computational model predicts phenotype from
genotype. Cell.

[btt036-B10] Kumar A (2012). MetRxn: a knowledgebase of metabolites and reactions spanning metabolic
models and databases. BMC Bioinformatics.

[btt036-B11] Rocha I (2010). OptFlux: an open-source software platform for in silico metabolic
engineering. BMC Syst. Biol..

[btt036-B12] Schellenberger J (2010). BiGG: a biochemical genetic and genomic knowledgebase of large scale
metabolic reconstructions. BMC Bioinformatics.

[btt036-B13] Terzer M (2009). Genome-scale metabolic networks. Wiley Interdiscip. Rev. Syst. Biol. Med..

